# The PI3K/AKT signalling pathway in inflammation, cell death and glial scar formation after traumatic spinal cord injury: Mechanisms and therapeutic opportunities

**DOI:** 10.1111/cpr.13275

**Published:** 2022-06-26

**Authors:** Xuegang He, Ying Li, Bo Deng, Aixin Lin, Guangzhi Zhang, Miao Ma, Yonggang Wang, Yong Yang, Xuewen Kang

**Affiliations:** ^1^ Department of Orthopedics Lanzhou University Second Hospital Lanzhou China; ^2^ The Second Clinical Medical College Lanzhou University Lanzhou China; ^3^ The International Cooperation Base of Gansu Province for the Pain Research in Spinal Disorders Lanzhou China; ^4^ Medical School of Yan'an University Yan'an University Yan'an China

## Abstract

**Objects:**

Traumatic spinal cord injury (TSCI) causes neurological dysfunction below the injured segment of the spinal cord, which significantly impacts the quality of life in affected patients. The phosphoinositide 3kinase/serine‐threonine kinase (PI3K/AKT) signaling pathway offers a potential therapeutic target for the inhibition of secondary TSCI. This review summarizes updates concerning the role of the PI3K/AKT pathway in TSCI.

**Materials and Methods:**

By searching articles related to the TSCI field and the PI3K/AKT signaling pathway, we summarized the mechanisms of secondary TSCI and the PI3K/AKT signaling pathway; we also discuss current and potential future treatment methods for TSCI based on the PI3K/AKT signaling pathway.

**Results:**

Early apoptosis and autophagy after TSCI protect the body against injury; a prolonged inflammatory response leads to the accumulation of pro‐inflammatory factors and excessive apoptosis, as well as excessive autophagy in the surrounding normal nerve cells, thus aggravating TSCI in the subacute stage of secondary injury. Initial glial scar formation in the subacute phase is a protective mechanism for TSCI, which limits the spread of damage and inflammation. However, mature scar tissue in the chronic phase hinders axon regeneration and prevents the recovery of nerve function. Activation of PI3K/AKT signaling pathway can inhibit the inflammatory response and apoptosis in the subacute phase after secondary TSCI; inhibiting this pathway in the chronic phase can reduce the formation of glial scar.

**Conclusion:**

The PI3K/AKT signaling pathway has an important role in the recovery of spinal cord function after secondary injury. Inducing the activation of PI3K/AKT signaling pathway in the subacute phase of secondary injury and inhibiting this pathway in the chronic phase may be one of the potential strategies for the treatment of TSCI.

## Graphical abstract

During secondary injury after spinal cord injury (SCI), recovery is impaired by inflammation, cell death and glial scar formation. Therefore, these pathological processes may be potential treatment targets for SCI. The phosphoinositide 3‐kinase/serine‐threonine kinase (PI3K/AKT) signalling pathway has a key role in the secondary injury after SCI. PI3K/AKT signalling pathway can inhibit the inflammation and cell death after SCI, and can also promote the formation of glial scar.

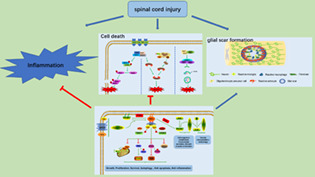

## 1 INTRODUCTION

**FIGURE 1:**
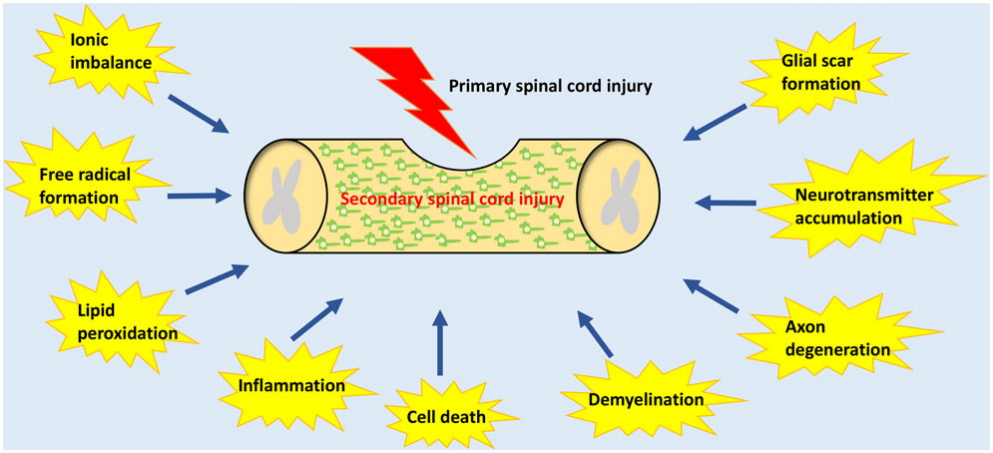
Summary of secondary injury processes following TSCI.

Spinal cord injury (SCI) is a serious neurological disease that is the second most common cause of paralysis after stroke.^1^ According to the Global Burden of Disease study, 27 million people (i.e., almost 368 per 100,000 people) worldwide experienced SCI between 1990 and 2016. In 2016, 4951 new patients experienced SCI (i.e., almost 13 per 100,000 people).^2^ The number of people affected by SCI is increasing annually. The main clinical manifestations of SCI include somatosensory deficits below the level of injury, voluntary movement paralysis, impaired sphincter contraction and diastolic function and autonomic dysfunction. The physical disability and high medical cost associated with SCI aggravate the mental health and economic condition of affected patients, leading to a high burden on the surrounding society and country.^3^ SCI may have traumatic or non‐traumatic causes. The non‐traumatic causes include spinal tumours, spinal stenosis, intervertebral disc herniation, myelitis, vascular malformations, infections and congenital spinal cord malformations.^4^, ^5^ The traumatic causes include traffic accidents, falls, sports injuries or violent trauma.^6^ Traumatic SCI (TSCI) may be caused by primary or secondary injury. Primary injury includes continuous compression or tearing of blood vessels and spinal cord tissue because of external forces, such as bone fragments, intervertebral discs or ligaments.^7^ Secondary injury includes ionic imbalance, free radical formation, lipid peroxidation, inflammation, cell death, demyelination, axon degeneration, excitatory neurotransmitter accumulation and glial scar formation^8^ (Figure 1). At present, the clinical treatment strategies for TSCI mainly include early surgical decompression, the maintenance of effective spinal cord blood perfusion, electrical stimulation, rehabilitation exercise and hormonal and neurotrophic factor therapies.^9^, ^10^, ^11^, ^12^ However, the aforementioned methods have limited efficacy. Experts recommend that surgery for spinal cord decompression after TSCI is accompanied by efforts to prevent secondary injury.^13^ Therefore, molecular targets should be identified that inhibit secondary injury.

Phosphoinositide 3‐kinase (PI3K) is a lipid kinase with key roles in physiological and pathological cellular activities. PI3K regulates cell proliferation, differentiation, programmed death and migration by activating the downstream target protein kinase B (known as PKB or AKT).^14^, ^15^ Excessive PI3K/AKT pathway activation in glioma promotes the proliferation, invasion and migration of glial cells.^16^ In stroke, PI3K pathway activation protects the neurons and brain from ischemic damage.^17^, ^18^, ^19^ In Parkinson's disease, PI3K/AKT pathway inhibition aggravates oxidative stress‐induced damage to dopamine neurons in the substantia nigra.^20^ In the acute kidney injury model, PI3K/AKT pathway activation inhibits apoptosis and inflammation.^21^ Multiple studies have shown that PI3K/AKT pathway activation after SCI prevents oxidative stress, inflammation and cell death, whereas PI3K/AKT pathway inhibition prevents the formation of glial scars.^22^, ^23^, ^24^, ^25^ Therefore, the PI3K/AKT pathway offers a potential therapeutic target for the inhibition of secondary SCI. This review summarizes updates concerning the role of the PI3K/AKT pathway in SCI.

## 2 INFLAMMATION, CELL DEATH AND GLIAL SCAR FORMATION IN SECONDARY SPINAL CORD INJURY

Secondary SCI begins within a few minutes after the primary injury and can last for several weeks or months, leading to progressive damage to the lesion and surrounding spinal cord tissue.^8^ Secondary injury can be divided into three different but overlapping continuous phases: acute, subacute and chronic.^8^, ^26^, ^27^ The acute phase lasts until 48 h after the primary injury, the subacute phase lasts until 2 weeks after the primary injury and the chronic phase can last until 6 months after the primary injury.^28^ The classification of secondary injury into these phases may be inaccurate and requires further studies. The acute phase begins immediately after the SCI and is mainly caused by mechanical damage, including vascular injury, ischemia, ionic imbalance, free radical formation and neurotransmitter accumulation.^29^, ^30^, ^31^ These injuries can continue into the subacute phase, which can last for a few weeks. The subacute phase, a continuation of the acute phase injury, includes lipid peroxidation, inflammatory response, cell death, axon demyelination and the beginning of glial scar formation.^30^, ^32^, ^33^, ^34^ In the chronic phase, vacuoles may appear at the injured site, surviving axons may undergo axotomy and glial scars may mature.^8^, ^35^ During the subacute phase after SCI, inflammation and cell death reduce the survival of peripheral nerve cells. In addition, a mature glial scar in the chronic phase inhibits axon regeneration after injury.^36^ The newly created encircling scar prevents axons from passing through the injury site to form an effective connection. The following section will describe the roles of inflammation, cell death and glial scar formation in secondary SCI to better understand the mechanisms of secondary injury and explore possible treatment measures.

### 2.1 Inflammation

Inflammation protects against tissue damage caused by trauma, ischemia, infection or toxins.^37^ A prolonged inflammatory response aggravates the damage to tissues and organs, thereby causing cell death.^38^ TSCI produces cell debris and releases intracellular proteins, which trigger an inflammatory response.^34^ This inflammatory response may be beneficial or harmful to the recovery after TSCI, depending on the timing after injury and activation state of immune cells.^8^, ^39^ Various cells are involved in the onset of inflammation after injury, including microglia, neutrophils, monocytes, macrophages and lymphocytes.

Microglia are resident immune cells (a type of macrophage) in the central nervous system that respond to early central nervous system damage.^40^ Microglia are the first cells to respond after SCI; they are activated within a few minutes and extend cytoplasmic protrusions toward the lesion.^40^ The morphology and function of activated microglia are similar to macrophages, which surround the injured site, prevent lesion expansion, remove cell debris and secrete anti‐inflammatory factors^41^, ^42^, ^43^; thus, activated microglia have a protective role. However, activated microglia and macrophages also release reactive oxygen species (ROS) and proinflammatory factors that cause secondary damage.^44^ Yang et al.^45^ found that 30 min after human SCI, microglia begin to release the inflammatory factors interleukin (IL)‐1β, IL‐6 and tumour necrosis factor α (TNF‐α). Nesic et al.^46^ showed that 72 h after SCI in rats, intrathecal injection of IL‐1 receptor antagonists significantly reduced injury‐induced apoptosis (rat spinal cord contusion model). In transgenic mice with TNF‐α overexpression, microglia and macrophages are activated, which cause the death of oligodendrocytes and release of myelin damage factors, ultimately leading to demyelination in mice.^47^ The above studies have shown that the proinflammatory cytokines IL‐1‐β and TNF‐α produced by microglia after SCI are involved in secondary injury.

In addition to the changes in microglial morphology and the release of chemokines and inflammatory factors, peripheral neutrophils and macrophages are recruited to the injured spinal cord.^48^ The first wave of infiltrating immune cells consists of neutrophils. In the first few hours after injury, neutrophils accumulate in the spinal cord from the peripheral blood, reaching peak level almost 1 day after injury; this is followed by a rapid decline.^49^, ^50^, ^51^, ^52^ At present, the role of neutrophils after SCI is controversial. Neutrophils help to ingest and remove tissue debris. Stirling et al.^53^ used lymphocyte antigen 6 complex locus (Ly6G) antibody to inhibit spinal cord neutrophil infiltration at 24 and 48 h after moderate Th9–Th10 contusion; they found that the reduction in neutrophil levels aggravated SCI in mice, suggesting that neutrophils have a beneficial role after SCI. However, many studies have shown that neutrophils release proinflammatory cytokines, chemokines, ROS and proteolytic enzymes at the injured site to activate other immune cells and glial cells, enhance the inflammatory cascade and aggravate injury; the inhibition of neutrophil infiltration is beneficial to the recovery of nerve function after spinal SCI.^40^, ^49^, ^50^, ^54^, ^55^, ^56^ Kubota et al.^57^ showed that myeloperoxidase can promote neutrophil infiltration, leading to increased apoptosis and decreased myelin retention after moderate Th9 contusion in mice. Saiwai et al.^58^ found that the inhibition of leukotriene B4 can inhibit white blood cell infiltration after SCI, thereby reducing inflammation and neuronal apoptosis, preserving spinal cord white matter and promoting neurological recovery (mice spinal cord contusion model). Bao et al.^59^ showed that the use of selective phosphodiesterase type 4 inhibitor reduced leukocyte infiltration, oxidative stress and neuropathic pain after thoracic‐clip‐compression SCI model in rat, while improving motor function.

After SCI, neutrophil infiltration decreases within 1 week, while there is increasing infiltration of macrophages derived from monocytes.^60^ Within 2–3 days after injury, the injured spinal cord is infiltrated by macrophages that are derived from blood monocytes or meninges and perivascular areas of the subarachnoid space.^61^, ^62^ The number of macrophages reaches a peak at 7–10 days after injury; this is followed by a gradual decrease. At 10 weeks after injury, 45% of the infiltrated macrophages remain in the lesion.^63^, ^64^ Macrophages have conflicting roles in the nervous system. Although they remove tissue debris, remodel the cytoskeleton and produce regenerative factors, they also release proteolytic enzymes, ROS and inflammatory factors, thus aggravating SCI.^48^, ^65^ Macrophages and microglia can be divided into M1 and M2 types on the basis of their phenotype and role in aggravating or repairing damage. The classically‐activated M1 phenotype has proinflammatory effects, including the release of ROS, IL‐1β, IL‐6, IL‐12, TNF‐α and other inflammatory factors or chemokines; these effects lead to the worsening of secondary injury.^66^, ^67^ Activated macrophages induce extensive retraction of dystrophic axons through direct physical interactions, whereas depletion of activated macrophages reduces axon retraction in a rat dorsal column crush lesion model.^68^ Classically activated microglia are also able to induce the activation of A1‐type reactive astrocytes, which amplify inflammation and produce neurotoxic effects, causing neuronal and oligodendrocyte death and glial scar formation.^69^ Chondroitin sulphate proteoglycans (CSPGs) inhibit axon growth. The level of CSPGs secreted by M1 macrophages is 16‐fold higher than the level of CSPGs secreted by M2 macrophages, suggesting that M1 macrophages hinder recovery after SCI.^70^, ^71^ The alternatively‐activated M2 phenotype has anti‐inflammatory effects and can be divided into M2a, M2b, M2c and M2d subtypes.^72^ The M2a subtype promotes tissue repair and regeneration by expressing anti‐inflammatory and immunomodulatory molecules.^73^ M2b microglia can be activated by Toll‐like receptors (TLRs), resulting in the expression of high levels of the anti‐inflammatory factor IL‐10 and low levels of IL‐12.^68^ The M2c subtype can be activated by IL‐10 and glucocorticoids, and highly express transforming growth factor beta (TGF‐β), and M2d microglia are ‘switched’ from a classically activated inflammatory phenotype to a selectively activated anti‐inflammatory phenotype type.^68^, ^74^ M2 phenotype can reduce ROS production and the secretion of anti‐inflammatory factors, such as IL‐4, IL‐10, insulin like growth factor‐1 (IGF‐1) and nutritional polyamines. M2 macrophages and microglia remove cell debris and stimulate tissue regeneration.^75^ In addition, studies have shown that microglia can also induce A2 astrocytes by downregulating P2Y1 purinergic receptor, which have neuroprotective effects and can promote tissue repair and regeneration.^76^ Although both M1 and M2 phenotypes are expressed after SCI, the M2 phenotype decreases rapidly after 1 week, while the M1 phenotype persists for up to 1 month,^64^ and the expression of M2a and M2c markers is positively correlated with A2 astrocytes.^77^ Therefore, shortening the duration of M1/A1 residence in the injured spinal cord and promoting polarization to the M2/A2 phenotype may inhibit secondary SCI.

Activated immune cells remove cell debris, prevent the spread of damage and secrete anti‐inflammatory factors, which control secondary injury. However, prolonged inflammation leads to the accumulation of proinflammatory factors, which damages normal nerve cells and aggravates SCI. Thus, there is a need to identify the favourable and harmful time points of the inflammatory response, suppress the activation of immune cells and inhibit the release of inflammatory factors during the harmful time period, and promote the polarization of macrophages and astrocytes to the anti‐inflammatory phenotype (M2/A2 phenotype).

### 2.2 Cell death

Cell death is an important cause of secondary injury to tissues surrounding the primary SCI. Cell death is the lysis of cells in an uncontrolled manner (i.e., necrosis), which causes cell contents to flow into the surrounding tissues or in a programmed manner through a series of biochemical and molecular pathways.^78^ The programmed cell death pathways identified by the Nomenclature Committee on Cell Death include regulated necrosis (necroptosis), extrinsic and intrinsic apoptosis, autophagic cell death and mitotic catastrophe.^79^ Here, we discuss the mechanism of programmed cell death after SCI, including apoptosis and two closely related pathways, necroptosis and autophagy.

**FIGURE 2:**
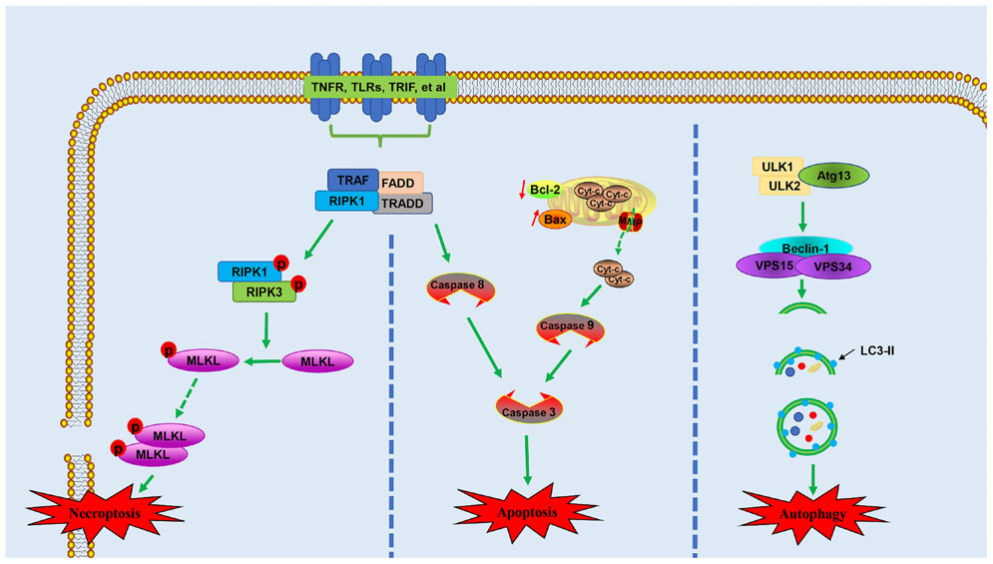
Schematic illustration of necroptosis, apoptosis and autophagy. Atg 13, autophagy‐related protein 13; Cyt‐c, cytochrome c; FADD, FAS‐associated death domain; LC3II, light chain 3 II; MLKL, mixed lineage kinase domain‐like protein; RIPK1, receptor‐interacting kinase 1; RIPK3, receptor‐interacting kinase 3; TLRs, toll‐like receptors; TNFR1, tumour necrosis factor receptor 1; TRADD, TNFR‐associated death domain; TRAF, tumour necrosis factor receptor associated factor; TRIF, toll interleukin 1 receptor‐domain‐containing adapter‐inducing interferon‐β; ULK 1, autophagy activating kinase 1; ULK 2, autophagy activating kinase 2

Apoptosis (also known as type I programmed cell death) of neurons and oligodendrocytes after SCI causes secondary injury.^80^ Therefore, a better understanding of apoptosis after SCI will help to identify new therapeutic strategies. During apoptosis, the nucleus condenses to form apoptotic bodies, which are eventually destroyed without causing inflammation. In a rat model of SCI, apoptosis begins 4 h after injury and peaks on day 7.^40^ Apoptosis is mediated by members of the caspase family; it can be induced by the extrinsic death receptor pathway or the intrinsic mitochondrial pathway.^81^ Excitatory toxins, free radicals and inflammatory mediators produced after SCI are the main factors that initiate apoptosis.^82^ The intrinsic apoptosis pathway is mainly mediated by apoptotic and anti‐apoptotic proteins on the mitochondrial membrane. When the apoptosis signal is received, the expression levels of apoptotic proteins Bax and Bad are increased, while the expression level of the anti‐apoptotic protein Bcl‐2 is decreased. In addition, the mitochondrial membrane potential is reduced; this causes the internal cytochrome c (Cyt‐c) and other apoptosis‐inducing factors to be released into the cytoplasm, where they form apoptotic bodies with deoxyadenosine triphosphate (dATP) and apoptosis protease‐activating factor‐1. The apoptotic bodies promote inactive procaspase‐9 to form activated caspase‐9; through the caspase cascade response activation system, this activated caspase‐9 activates procaspase‐3 to form activated caspase‐3 (Figure 2).^83^, ^84^ This process leads to DNA damage, destruction of both nucleoproteins and the cytoskeleton, expression of phagocyte ligands and formation of apoptotic bodies, which are then phagocytosed by immune cells.^85^ Members of the caspase recruitment domain family have been shown to reduce the mitochondrial release of Cyt‐c into the cytoplasm and inhibit the expression of caspase‐3, thus inhibiting apoptosis after SCI.^86^ The extrinsic apoptotic pathway is also known as the death receptor pathway, which is initiated by the binding of a death ligand to the death receptor on the cell membrane. Death receptors belong to the TNF superfamily; they include TNF receptor 1 (TNFR1), Fas (also known as CD95 and APO‐1), death receptor 3 (DR3) and TNF‐related apoptosis‐inducing ligand receptor 1 (TRAIL‐R1, also known as DR4), as well as the corresponding death ligands, such as TNF, Fas‐ligand (Fas‐L, also known as CD95‐L), TLIA and TRAIL (also known as Apo2‐L). After these ligands bind to their respective death receptors, procaspase‐8 is recruited to the death‐inducing signal complex bound to the death receptors on the cell membrane via the death‐inducing domain of procaspase‐8, thereby activating caspase‐8. The death‐inducing signal complex contains FAS‐associated death domain‐ or TNFR‐associated death domain‐binding proteins, which promote the interaction between caspase‐8 and the death‐inducing signal complex.^87^, ^88^, ^89^ The activated caspase‐8 activates caspase‐3, thereby causing apoptosis^90^ (Figure 2). Fas‐deficient mice have been shown to exhibit reduced levels of neuronal and oligodendrocyte apoptosis after SCI, along with decreases in both macrophage infiltration and inflammatory cytokine expression; furthermore, these mice exhibit significant improvement in their motor function.^91^

Necrosis was originally regarded as unprogrammed cell death. When necrosis is stimulated by hypoxia or external physical/chemical damage, the permeability of the necrotic cell membrane increases, causing cell swelling, organelle enlargement or deformation and finally cell rupture. The cell contents overflow into the surrounding area, which triggers inflammation and tissue damage.^89^ The external force during SCI can directly lead to neuronal necrosis. During secondary injury, hypoxia, ATP depletion, electrolyte imbalance, free radicals, inflammatory factors and toxic neurotransmitters related to vascular injury cause swelling and necrosis in surrounding nerve cells. In recent years, a new type of cell death has been discovered, with characteristics similar to necrosis: this process, necroptosis or regulated necrosis, is triggered by the binding of death ligand to the death receptor.^92^ During necroptosis, a complex is formed that comprises TNFR‐associated death domain, FAS‐associated death domain, receptor‐interacting kinase 1 (RIPK1) and TNFR‐associated factors.^93^ When caspase‐8 activity is inhibited, RIPK1 can combine with RIPK3 through the RIP homotypic interaction motif of the C‐terminal domain to form an amyloid complex known as the necrosome. Then, RIPK3 is activated by phosphorylation at Ser345; this activated RIPK3 phosphorylates the mixed lineage kinase domain‐like protein (MLKL). Phosphorylated MLKL triggers the formation of oligomers of phosphatidylinositol lipids and cardiolipin, which allows MLKL to transfer from the cytoplasm to the cell membrane and form a hole in the membrane, thereby destroying plasma membrane integrity and causing necroptosis^94^ (Figure 2). Necroptosis is an important type of cell death after SCI; the inhibition of necroptosis may be effective for treating SCI. The use of necrostatin‐1 (Nec‐1) to treat SCI mice has been shown to significantly reduce the number of cells with loss of plasma membrane integrity in spinal cord tissue; it also significantly reduced the size of the injured area.^95^ Additionally, Nec‐1 alleviated necroptosis in nerve cells after SCI by inhibiting the recruitment of RIPK1/3‐MLKL; this promoted the recovery of motor function.^96^

Autophagy is a self‐protective mechanism of cells, a cytoprotective response activated by cells to cope with stress.^79^ Autophagy involves the degradation of damaged proteins, organelles and other cellular materials via lysosomes under conditions of nutrient deficiency or hypoxia. These degradation products enter metabolic and biosynthetic pathways or are oxidized by mitochondrial ATP for cell survival.^97^, ^98^ Based on the method of substrate entry into the lysosome, autophagy can be divided into chaperone‐mediated autophagy, microautophagy and macroautophagy. In chaperone‐mediated autophagy, cells interact with the lysosomal protein LAMP‐2A via recognition of a common motif in the chaperone‐mediated autophagy substrate sequence; they then transfer intracellular proteins to the lysosome, which results in the degradation of intracellular proteins. Microautophagy refers to invagination of the lysosomal membrane for the phagocytosis of cytoplasmic substrates. Macroautophagy is characterized by the formation of autophagosomes with a double‐layered membrane structure; these autophagosomes fuse with lysosomes to form autophagolysosomes, which degrade and recycle the phagocytosed substances.^99^, ^100^ Macroautophagy is the most common form of autophagy (hereinafter, macroautophagy is referred to as autophagy); it can be activated by unc‐51‐like autophagy activating kinase 1 (ULK 1), ULK 2, autophagy‐related protein 13 (Atg13) and Beclin‐1/III PI3K complexes. When autophagy is activated, these complexes recruit other proteins involved in the extension and formation of autophagosomes (e.g., Atg12, Atg5, Atg16L and light chain 3 [LC3]) to form conjugate complexes. LC3 combines with phospholipid–phosphatidylethanolamine to form LC3II. LC3II enters the autophagosome membrane, causing autophagosomes to extend and mature. Mature autophagosomes fuse with lysosomes to form autophagosomes, in which isolated substances and organelles are degraded by lysosomal enzymes.^101^ The mechanistic target of rapamycin complex 1 (mTORC1) is regarded as a negative regulator of autophagy; it inhibits autophagy via phosphorylation of Atg.^102^ Inhibition of mTORC1 leads to dephosphorylation and activation of the ULK complex, which activates class III PI3K to promote autophagy^103^ (Figure 2). Recent studies have shown that autophagy has a neuroprotective effect after SCI.^104^ Kanno et al.^105^ first reported the overexpression of autophagy marker proteins Beclin‐1 and LC3II around experimental SCI lesions. The level of Beclin‐1 expression is upregulated beginning at 4 h after injury, reaches a peak on the third day, and lasts for 21 days after SCI. Autophagy biomarkers also increase in human central nervous system damage; this increase lasts for several weeks or months.^106^ The long‐term activation of autophagy may reflect its neuroprotective effect on secondary injury. He et al.^107^ showed that autophagy increased the stability of microtubules by degrading the microtubule destabilizing protein SCG10; it promoted axon regeneration after SCI. Li et al.^108^ found that the inhibition of bromodomain protein 4 after SCI enhanced neuronal autophagy and restored autophagic flux, thereby reducing oxidative stress and neuronal apoptosis; it also promoted functional recovery. The Atg1 kinase complex is necessary to activate autophagy; mTORC1 inhibits autophagy by inhibiting the Atg1 kinase complex.^109^ Use of the mTOR inhibitor rapamycin effectively increases the expression levels of LC3II and Beclin1, inhibits apoptosis and promotes the recovery of nerve function after SCI.^110^ In addition, disruption of autophagic flow also makes it difficult to recover axonal stumps after injury. Studies have shown that CSPG, through protein tyrosine phosphatase receptor sigma (PTPR σ), leads to dephosphorylation of cortactin, disruption of autophagy flux, dystrophic endball formation and, ultimately, inhibition of axonal regeneration.^111^ Although autophagy is generally regarded as a protective response, excessive autophagy may lead to cell death. The inhibition of excessive autophagy has been shown to improve neurological function in SCI rats.^112^ Moderate activation of autophagy promotes cell survival, whereas excessive activation leads to cell death. Autophagy is a dynamic process that has positive or negative effects, depending on its timing.^113^ The promotion of autophagy immediately after ischemic SCI promotes the recovery of motor function, while the inhibition of autophagy has the opposite effect; inhibition of autophagy at 48 h after SCI increases the recovery of motor function, while stimulation of autophagy has no effect.^114^ Therefore, autophagy at an early stage (i.e., immediately after injury) after SCI inhibits apoptosis and inflammation; however, excessive autophagy at a later stage (i.e., 48 h after injury) aggravates the injury by inducing autophagic cell death.

Various direct and indirect interactions exist between autophagy and apoptosis, suggesting that a coordinated balance between autophagy and apoptosis regulatory proteins determines the cell fate.^115^ Autophagy and apoptosis usually occur in the same cell, with autophagy preceding apoptosis.^116^ This is because stress, particularly non‐fatal stress, usually stimulates the autophagy response. When the stress level exceeds a critical time or intensity threshold, apoptotic or non‐apoptotic lethal programs are activated.^117^ Autophagy is often a strategy for adapting and coping with stress. Autophagy enables lysosomes to degrade damaged mitochondria and dysfunctional cell components, thus preventing apoptosis.^118^ The selective autophagic degradation of p53 upregulated modulator of apoptosis (PUMA) prevents an increase in mitochondrial membrane permeability and slows the process of apoptosis.^119^ Autophagy can selectively degrade caspase‐8 and inhibit TRAIL‐induced apoptosis.^120^ In contrast, apoptosis can inhibit the process of autophagy, thus leading to increases in caspase levels. Caspases can digest several important autophagy proteins, leading to inactivation of the autophagy program. The carboxy‐terminal fragments of Beclin‐1, formed by caspases‐3 and ‐9, are localized to the mitochondrial membrane; this localization increases the mitochondrial membrane permeability and promotes Cyt‐c release, leading to apoptosis.^117^ Therefore, the inhibition of cell death during the subacute phase of secondary injury promotes recovery after SCI. The inhibition of nerve cell apoptosis to activate autophagy or the inhibition of excessive autophagy, may protect the surrounding tissues from damage and prevent the development of secondary injury. Further research is needed to enhance the general understanding of the mechanisms underlying apoptosis, necroptosis and autophagy after SCI, then identify the roles of such processes (e.g., at different times and in different cell types).

### 2.3 Glial scar formation

**FIGURE 3:**
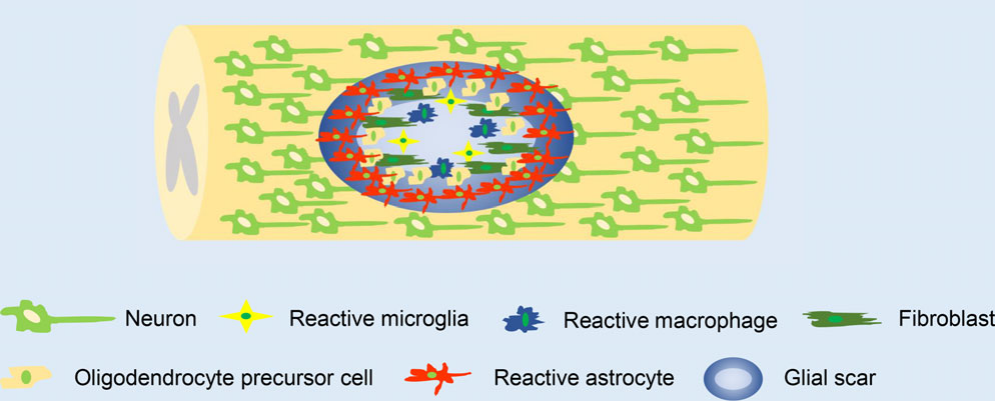
Schematic illustration of glial scar formation in chronic phase of secondary spinal cord injury.

After the primary injury, inflammatory cells infiltrate into the injured tissue and release inflammatory factors, chemokines and enzymes. In addition, astrocytes, oligodendrocyte precursor cells and fibroblasts are activated, which causes them to undergo hypertrophy, thickening and migration to the centre of the lesion.^121^, ^122^ Reactive astrocytes proliferate and express intermediate filaments, glial fibrillary acidic protein, nestin and vimentin; this process leads to the formation of glial scars composed of a network of interwoven filamentous protrusions around the injury centre.^36^ These astrocytes show the fastest proliferation 3–5 days after injury, followed by slowed proliferation 7 days after injury and a near absence of proliferation 14 days after injury.^123^ Glial scars have dual roles after SCI. Astrocyte proliferation appears to be a protective mechanism. Scar tissue produces growth‐promoting substances, such as fibronectin and laminin. In addition, newly forming glial scars limit and repair local damage by isolating the diseased area, restricting the spread of inflammation, producing growth factors and restoring the blood‐spinal cord barrier.^124^, ^125^, ^126^ The administration of ganciclovir to eliminate the reactive astrocytes in SCI mice in the acute or subacute phase has been shown to cause blood–brain barrier repair disruption, increased leukocyte infiltration, local tissue destruction, severe demyelination, neuron and oligodendrocyte death and movement disorders.^127^ Prevention of glial scar formation at an early stage after SCI significantly increases axon death, rather than causing spontaneous regeneration of transected axons.^128^ The aforementioned studies have shown that the prevention of early glial scar formation does not improve repair after SCI. However, the formation of glial scars creates a physical barrier for neuronal regeneration and axon repair. Between 1–2 and several weeks after injury, the glial scar matures.^129^ The scar persists for an extended period of time; scars have been found in humans almost 42 years after SCI.^130^ In mature glial scars, activated microglia and macrophages are located on the innermost side of the injury centre, surrounded by oligodendrocyte precursor cells; reactive astrocytes are located in the injury penumbra, where they form a cellular barrier^130^ (Figure 3). Early studies found that regenerated axons cannot grow through the glial scars, suggesting that glial scars form a physical barrier that hinders axon regeneration.^131^, ^132^ A study of SCI in rats and dogs found a clear boundary between normal tissue and glial scar tissue outside of the injured cavity at 4 weeks after injury; only a few axons were located in the outer and inner layers of the glial scar.^133^ These findings indicate that axons have the potential to regenerate after injury, but the regeneration is prevented by glial scars. In addition to a mechanical barrier, the glial scar contains various chemicals that inhibit axon growth, thus creating a chemical barrier. After SCI, the expression levels of intermediate filaments, such as glial fibrillary acidic protein and vimentin, are upregulated. Knockout experiments have shown that the absence of these filaments can reduce glial scar formation and promote axon growth after central nervous system injury.^134^, ^135^ CSPG is a proteoglycan family characterized by chondroitin sulphate side chains, which are mainly secreted by reactive astrocytes and combined with other components of the extracellular matrix (including collagens, tenascins, semaphorins and ephrins) in glial scars. CSPGs are key inhibitors of axon regeneration in the central nervous system.^35^, ^136^ The expression of CSPGs in the penumbra of the lesion changes over time; the level of CSPG‐glycosamine polysaccharides is at least four‐fold greater in chronic glial scars than in subacute glial scars.^137^ In addition, targeted inhibition of CSPGs can enhance oligodendrocyte differentiation and remyelination after SCI.^138^, ^139^ CSPGs inhibit axon growth through PTPR σ.^70^, ^140^ Knockout experiments have shown that the absence of the *PTPRS* gene, which encodes PTPR σ, significantly enhances axon regeneration after SCI.^141^ The enzyme chondroitinase ABC (Ch ABC) degrades the glycosaminoglycan chain of CSPGs, attenuates the inhibitory activity of CSPGs and promotes axon regeneration.^142^

Based on the aforementioned evidence, glial scars formed at different time points after SCI may have protective or harmful roles in the clinical recovery. In the acute and subacute phases, glial scars limit the expansion of the damage and protect the remaining spinal cord tissue; however, in the chronic phase, mature glial scars form physical and chemical barriers, which hinder axon regeneration and prevent the recovery of nerve function. Therefore, the removal of glial scars in the chronic phase, rather than the acute or subacute phase, may offer a treatment strategy for spinal cord injury.

## 3 PI3K/AKT SIGNALLING PATHWAY

**FIGURE 4:**
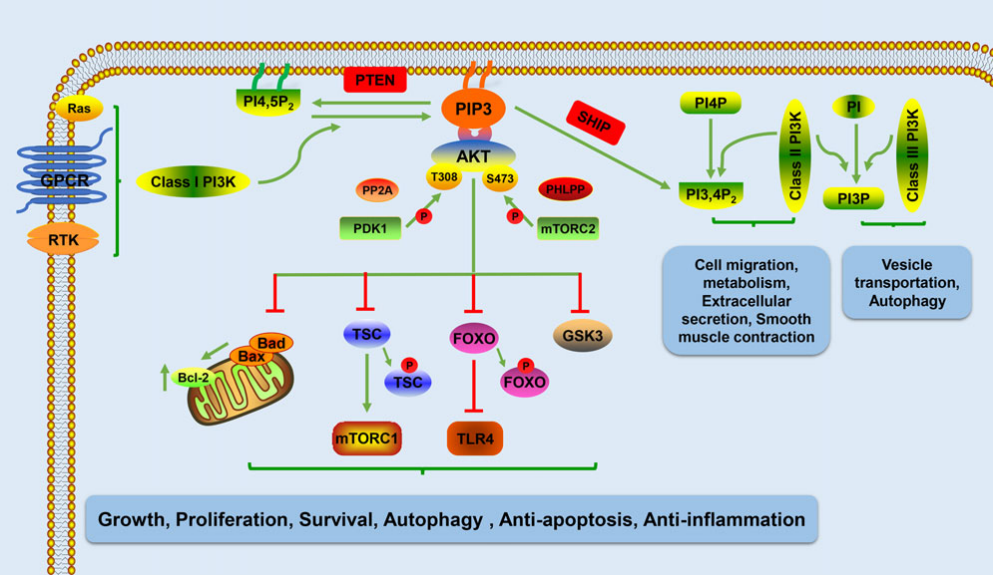
Schematic illustration of the PI3K signalling pathway. AKT/PKB, protein kinase B; FOXO, forkhead box O; GPCR, G protein‐coupled receptors; GSK3, glycogen synthase kinase 3; mTORC1, mechanistic target of rapamycin complex 1; mTORC2, mechanistic target of rapamycin complex 2; PDK 1, phosphoinositide‐dependent protein kinase 1; PHLPP, PH domain leucine‐rich repeat protein phosphatases; PI, phosphatidylinositol; PI3,4P2, phosphatidylinositol 3,4‐bisphosphate; PI3K, phosphoinositide 3‐kinase; PI3P, phosphatidylinositol 3‐phosphate; PI4,5P2, phosphatidylinositol 4,5‐bisphosphate; PI4P, phosphatidylinositol 4‐phosphate; PIP3, phosphatidylinositol 3,4,5‐trisphosphate; PP2A, protein phosphatase 2A; PTEN, phosphatase and tensin homologue deleted on chromosome ten; RTK, receptor tyrosine kinases; SHIP, SH2‐domain‐containing inositol 5‐phosphatase; TLR4, toll‐like receptor 4; TSC, tuberous sclerosis complex

The PI3K family is divided into three categories (classes I–III) based on differences in primary structure and specificity for lipid substrates. Class I PI3Ks have been studied extensively. In mammals, class I PI3Ks exist in all cell types and are activated by receptor tyrosine kinases, G protein‐coupled receptors and Ras family proteins on the cell membrane.^143^, ^144^ The activated class I PI3Ks convert phosphatidylinositol 4,5‐bisphosphate (PI4,5P2) into the second messenger phosphatidylinositol 3,4,5‐trisphosphate (PIP3).^145^, ^146^ Phosphatase and tensin homologue deleted on chromosome ten (PTEN) prevents this conversion by dephosphorylation at the D3 position of PIP3 and subsequent hydrolysis to produce the inactive PI4,5P2.^143^ SH2‐domain‐containing inositol 5‐phosphatase also dephosphorylates PIP3 to produce PI3,4P2.^147^ PIP3 and PI3,4P2 regulate the positions and functions of multiple effector proteins, which bind to lipids through their PH domains. These include serine/threonine and Tyr protein kinases (such as AKT and BTK, respectively), junction proteins (such as GAB2) and regulatory proteins of small GTPases (e.g., GAP and GeFs).^143^ Studies of class I PI3K signalling pathways have mainly focused on AKT and its downstream targets. PIP3 activates AKT by phosphorylating the threonine phosphorylation site (Thr308) and serine phosphorylation site (Ser473) on the AKT protein. Phosphoinositide‐dependent protein kinase 1 phosphorylates the T308 site, which is necessary for AKT activation.^148^ Maximum activation of AKT requires S473 phosphorylation in the hydrophobic group, which involves mTORC2. Although AKT without S473 phosphorylation is active, it has significantly less activity. S473 phosphorylation stabilizes T308 phosphorylation and AKT activation.^149^, ^150^ Protein phosphatase 2A (PP2A) can dephosphorylate T308 in AKT and inactivate kinases; in contrast, PH domain leucine‐rich repeat protein phosphatases 1 (PHLPP1) and PHLPP2 are responsible for the physiological dephosphorylation of AKT S473, thereby terminating the AKT phosphatase signal.^151^, ^152^ AKT activation directly activates or inhibits (through phosphorylation) the downstream protein targets, including protein and lipid kinases, transcription factors, small G proteins, vesicle transport regulators, metabolic enzymes, E3 ubiquitin ligase and cell cycle regulators.^147^ For instance, AKT activation inhibits glycogen synthase kinase 3 (GSK3) and forkhead box O family (FOXO) transcription factors; it activates mTORC1 by inhibiting the tuberous sclerosis complex. These effects contribute to growth, translation, cell cycle regulation, glucose metabolism, cell autophagy, DNA repair, anti‐inflammatory effects and apoptosis inhibition^153^, ^154^, ^155^ (Figure 4).

Class II PI3Ks were identified on the basis of sequences homologous to classes I and III PI3Ks, instead of their functions. The mechanism of action of class II PI3Ks is poorly understood and unclear. Class II PI3Ks are mainly present in the intracellular membrane and may be expressed in the nucleus, but they are only present at low levels in the cytoplasm.^143^ Class II PI3Ks catalyse the phosphorylation of phosphatidylinositol 4‐phosphate (PI4P) to form PI3,4P2, which directly or indirectly recruits proteins that cause actin filament rearrangement and membrane deformation.^156^ Class II PI3Ks also catalyse the conversion of PI to PI3P.^157^ Studies of mammalian cells have shown that class II PI3Ks are involved in cellular processes such as migration, glucose metabolism, extracellular secretion, smooth muscle cell contraction and apoptosis^158^ (Figure 4).

Class III PI3Ks (e.g., Vps34) were first discovered in yeast. The function of class III PI3Ks is highly conserved from yeasts to mammals; these proteins have key roles in diverse cellular processes.^159^ Vps34 and its regulatory protein kinase subunit Vps15 form a complex that catalyses the conversion of phosphatidylinositol to PI3P, which regulates endosome transport and autophagosome formation^160^, ^161^ (Figure 4).

## 4 PI3K/AKT SIGNALLING PATHWAY IN SECONDARY SPINAL CORD INJURY

Many studies have evaluated the roles of the class I PI3K/AKT signalling pathway in secondary SCI, such as the regulation of cell growth and the control of anti‐inflammatory and anti‐apoptosis effects. Here, we discuss the complex roles of the class I PI3K/AKT signalling pathway in secondary injury, mainly related to inflammation, cell death and glial scar formation.

### 4.1 PI3K/AKT signalling pathway regulates inflammation

The PI3K/AKT signalling pathway has an important role in regulating the inflammatory response after SCI. After SCI, damage‐associated molecular patterns (e.g., myelin, cell debris, various proteins and ATP) activate TLRs on inflammatory cells, thus triggering inflammation.^162^, ^163^, ^164^ Activation of the PI3K/AKT signalling pathway has been shown to inhibit TLR4 expression and reduce the release of inflammatory factors (e.g., TNF‐α and IL‐6^165^) from lipopolysaccharide (LPS)‐stimulated microglia. Activated AKT phosphorylates the transcription factor FOXO1, thereby promoting the nuclear translocation of FOXO 1, exportation of FOXO 1 from the nucleus to the cytoplasm, inhibition of both FOXO 1 transcriptional activity and the expression of its target gene TLR4, and reduction of inflammation.^166^, ^167^ NF‐κB is a classic transcription factor for many proinflammatory cytokines. The TLR4 receptor promotes the polarization of macrophages and microglia to the M1 phenotype, as well as the expression of proinflammatory factors (e.g., TNF‐α, IL‐1β and IL‐6), by activating NF‐κB.^168^, ^169^ LPS has been shown to induce PI3K/AKT pathway activation and negatively regulate NF‐κB, thus reducing the expression of TNF‐α in monocytes. Therefore, inhibition of the PI3K/AKT pathway can enhance the gene expression of LPS‐induced TNF‐α and increase p65 nuclear translocation.^170^ Activated AKT activates mTOR by phosphorylation and inactivation of the tuberous sclerosis complex. The inhibition of tuberous sclerosis complex 2 (TSC2), a key negative regulator of mTOR, inhibits NF‐κB expression, enhances STAT3 activity and reduces the transfer of proinflammatory cytokines.^171^ The regulation of NF‐κB by the PI3K/AKT signalling pathway is controversial. Many studies have shown that the PI3K/AKT pathway positively regulates NF‐κB and promotes inflammation.^172^, ^173^ In the cytoplasm, the NF‐κB inhibitor IκB is negatively regulated in the inactive state. Activated AKT has been shown to phosphorylate IκB kinase (IKK), enhance IKK activity and increase the nuclear transduction of NF‐κB, thus promoting the expression of inflammatory factors.^172^ In human monocytes, the use of rapamycin inhibits the mTOR pathway, thereby inhibiting the anti‐inflammatory effect of dexamethasone and leading to the continuous production of proinflammatory cytokines.^174^ The PI3K/AKT signalling pathway is involved in the process of macrophage polarization. During inflammation in humans, PI3K/AKT pathway activation changes microglia polarization from the proinflammatory M1 phenotype to the anti‐inflammatory M2 phenotype; this significantly increases the expression levels of IL‐1ra, IL‐10 and interferon‐β, while significantly decreasing the expression levels of proinflammatory cytokines (IL‐1, TNF‐α, IL‐6, IL‐8 and CXCL1).^175^ In addition, mTOR also affects the polarization of macrophages. After using rapamycin to inhibit mTOR, M1 type macrophages release more inflammatory factors (IL‐1β, TNF‐α and IL‐6), while M2 type macrophages increase apoptosis.^176^

The aforementioned studies have shown that the PI3K/AKT signalling pathway plays an important role in regulating the inflammatory response in SCI. PI3K/AKT pathway activation inhibits the activation of immune cells, as well as the release of proinflammatory cytokines. In addition, PI3K/AKT pathway activation may decrease the expression of proinflammatory cytokines by inhibiting the transcription factor NF‐κB. This pathway also reduces the conversion of macrophages to the inflammatory phenotype and limits the secretion of anti‐inflammatory factors. Therefore, activation of the PI3K/AKT signalling pathway via inflammation inhibition offers a potential treatment strategy for SCI.

### 4.2 PI3K/AKT signalling pathway regulates cell death

The PI3K/AKT pathway has an important role in regulating programmed cell death. PI3K/AKT pathway activation during necroptosis inhibits MLKL activation and necroptosis.^177^ Phosphorylated AKT inhibits the RIP1/RIP3/MLKL pathway and necroptosis induced by hydrogen peroxide.^178^ Selenomethionine is an organic source of selenium that is widely used in food supplements. The use of selenomethionine in kidney injury models inhibits the key molecules of necroptosis (RIP1, RIP3 and MLKL) by activating the PI3K/AKT pathway.^179^ The aforementioned studies showed that PI3K/AKT pathway activation inhibits necroptosis. However, other studies have shown conflicting results. Hu et al.^180^ showed that PI3K binds to RIP3, but not RIP1, to form protein complexes and mediate TNF‐induced necroptosis without RIP1. These findings indicate that PI3K is essential for TNF‐induced necroptosis and may act with RIP3 to initiate activation of the RIP1–RIP3–MLKL signalling pathway and subsequent necroptosis. These conflicting results may be explained by differences in experimental models and diseases between studies. Thus far, no study has evaluated the effect of the PI3K/AKT pathway on necroptosis in SCI models and follow‐up studies are needed.

PI3K/AKT is a key signal pathway involved in the regulation of apoptosis. PI3K/AKT signalling pathway activation reduces the expression levels of caspase‐9 and ‐3; it inhibits apoptosis after SCI in rats.^181^ Phosphorylated AKT phosphorylates the apoptotic protein Bad and eliminates its counterpart. The apoptotic protein Bcl‐XL blocks the Bax‐mediated formation of channels on the mitochondrial membrane, thus inhibiting Cyt‐c release and apoptosis.^182^ Li et al.^183^ showed that PI3K/AKT signalling pathway activation inhibited LPS‐induced apoptosis in endothelial cells, thereby preventing destruction of the brain‐spinal cord barrier after SCI and slowing the progression of secondary injury. Jung et al.^184^ found that treadmill exercise promoted the expression of rat neurotrophic factors (NGF, NT‐3 and IGF‐1), activated the PI3K/AKT pathway, inhibited spinal cord apoptosis and improved motor function after SCI. In a rat SCI model, PI3K/AKT pathway activation downregulated the levels of Fas, FasL and caspase; inhibited apoptosis via the extrinsic pathway; and increased the production of cytoskeletal proteins. In addition, α‐tubulin, microtubule‐associated protein 2 and neurofilament protein‐200 contribute to the retention of structural integrity and promote the recovery of motor function.^185^ The PI3K/AKT signalling pathway is activated in the medulla after medullary injury, which leads to inactivation of the apoptosis‐inducing factor FKHR (a downstream target of p‐AKT). The PI3K inhibitor LY294002 reduces the level of damage‐induced AKT. FKHR phosphorylates and activates FasL in the medulla oblongata, promotes apoptosis and inhibits the recovery of spontaneous breathing.^186^

As mentioned previously, PI3K/AKT signalling pathway activation inhibits apoptosis. In theory, PI3K/AKT signalling pathway activation promotes autophagy. However, multiple studies have shown contradictory results. The autophagy inhibitor mTORC1 is activated by phosphorylated AKT; therefore, inhibition of the PI3K/AKT/mTOR pathway may promote autophagy.^187^ Wang et al.^188^ showed that the injection of LY294002 in SCI rats decreased AKT phosphorylation, mTOR and apoptosis levels, while it increased the LC3II/I ratio and the proportion of cells undergoing autophagy. Chen et al.^189^ showed that inhibition of the PI3K/AKT/mTOR pathway increased the LC3II/I ratio, downregulated the expression levels of proapoptotic proteins (Bax, caspase‐3 and caspase‐9) and upregulated the expression levels of Beclin‐1 and the anti‐apoptotic protein Bcl‐2. These results indicate that inhibition of the PI3K/AKT/mTOR pathway increased autophagy, inhibited apoptosis and promoted the recovery of nerve function after SCI; thus, inhibition of the PI3K/AKT/mTOR pathway may be a potential treatment strategy for SCI.

Inhibition of mTORC1, a downstream target of the PI3K/AKT signalling pathway, promotes autophagy and inhibits apoptosis. However, activation of the PI3K/AKT signalling pathway may have anti‐apoptotic effects. Further research is needed to determine whether activation or inhibition of the PI3K/AKT signalling pathway has anti‐apoptotic effects. To produce rigorous results, future research should use models to modify the doses of activators or inhibitors of the PI3K/AKT signalling pathway, effects on other signalling pathways, durations of action, time points to detect the impact on apoptosis or autophagy and cell types.

### 4.3 PI3K/AKT signalling pathway regulates glial scar formation

PI3K/AKT signalling pathway activation is the main determinant of glial scar formation^190^; inhibition of this pathway can reduce glial scar formation.^191^ Inhibition of mTOR activation has been shown to reduce astrocyte proliferation and inhibit the cell cycle, thereby reducing the formation of glial scars after SCI.^192^ CSPGs are important components of extracellular matrix in glial scar tissues; they limit axon regeneration after SCI.^193^ Chen et al.^191^ found that PTEN expression in the spinal cord regulates the expression patterns of CSPGs. In addition, PTEN overexpression causes astrocytes to activate the PI3K/AKT/mTOR pathway, increase the expression of intermediate filament proteins (glial fibrillary acidic protein and vimentin) and reduce the expression of proliferating cell nuclear antigen (PCNA, a proliferation‐promoting protein). PTEN overexpression also reduced the expression of CSPGs, prevented the growth of glial scars, promoted axon growth and enhanced the recovery of nerve function in SCI rats. Therefore, the PI3K/AKT/mTOR signalling pathway is involved in the formation of glial scars. Fibronectin is a ubiquitous, abundant extracellular matrix protein, which is secreted in the form of soluble dimers that are assembled into multimeric fibrils on the cell surface.^194^ Fibronectin is also a component of glial scars; it can increase the expression of the P2Y1 receptor (present in astrocytes) via AKT phosphorylation and it enhances the proliferation of spinal cord astrocytes. Fibronectin activates AKT through the heterodimeric α5β1 integrin receptor; siRNA‐mediated changes in α5β1 integrin block the phosphorylation of AKT by fibronectin.^195^ TGF‐β is one of several cytokines involved in glial scar formation and CSPG expression; TGF‐β induces the expression of CSPGs through the activation of the PI3K/AKT pathway. TGF‐β inhibition can reduce glial scar formation after central nervous system injury.^196^ In general, activation of the PI3K/AKT signalling pathway promotes the proliferation of spinal cord astrocytes, whereas inhibition of this pathway reduces the formation of glial scars and promotes recovery after SCI.

## 5 REGULATION OF PI3K/AKT SIGNALLING PATHWAY IN SPINAL CORD INJURY

PI3K/AKT, a classical signalling pathway, has an important role in secondary SCI and is therefore a potential therapeutic target. Numerous studies have shown that activators or inhibitors of the PI3K/AKT signalling pathway can be used to treat SCI. Here, we discuss the roles of these factors in the treatment of SCI through regulation of the PI3K/AKT signalling pathway, including non‐coding RNA, biomolecules and drugs.

### 5.1 Non‐coding RNA

According to the Human Genome Project, less than 2% of the RNA sequences generated by human DNA can be translated into proteins. Most of the remaining RNA sequences cannot be translated and are known as non‐coding RNAs (ncRNAs). Although ncRNAs cannot be transcribed into proteins, they have indispensable roles in the growth and development of organisms through their effects on transcription and translation. Abnormal expression of ncRNAs after SCI has been shown to affect the development of secondary injury.^197^ Below, we review the ncRNAs associated with the PI3K/AKT signalling pathway after SCI, including microRNAs (miRNAs), long non‐coding RNAs (lncRNAs) and circular RNAs (circRNAs) (Table 1).

**TABLE 1:** Non‐coding RNAs associated with the PI3K/AKT signalling pathway after SCI

ncRNAs	Expression after SCI	Target­genes/miRNAs	Mechanisms	Functions	References
*miRNAs*					
miR‐21‐5p	Upregulated	—	miR‐21‐5p ↑→ p‐PI3K, p‐AKT↑, PTEN↓, Bax↓, Bcl‐2↑, Caspase‐3↓, Caspase‐9↓, IL‐8↓, IL‐1β↓, IL‐6↓, TNF‐α↓	Anti‐apoptosis and anti‐inflammatory	[198]
miR‐138‐5p	Upregulated	Sirtuin 1	miR‐138‐5p ↓→ Sirtuin 1 ↑→ p‐AKT↑, PTEN↓, IL‐1β↓, IL‐6↓, TNF‐α↓	Promote apoptosis and inflammation	[199]
miR‐135a‐5p	Downregulated	SP1, ROCK	miR‐135a‐5p ↑→ SP1↓, ROCK ↓→ p‐AKT↑, p‐GSK3β↑, Bax↓, Bcl‐2↑, Caspase‐3↓,	Promote apoptosis and suppress axon regeneration	[200]
miR‐338‐5p	Downregulated	Cnr1	miR‐338‐5p ↑→ Cnr1 ↓→ Rap1 ↑→ p‐PI3K↑, p‐AKT↑, Bax↓, Bcl‐2↑, Caspase‐3↓	Promote apoptosis	[201]
miR‐122‐5p	Downregulated	CPEB1	miR‐122‐5p ↑→ CPEB1 ↓→ p‐PI3K↑, p‐AKT↑, IL‐6↓, TNF‐α↓, IL‐1β↓, IL‐10↑, Bax↓, Bcl‐2↑, Caspase‐3↓	Promote apoptosis and inflammation	[202]
miR‐34a	Downregulated	CD47	miR‐34a ↑→ CD47 ↓→ p‐PI3K, p‐AKT↑, Bax↓, Bcl‐2↑, Caspase‐3↓	Promote apoptosis	[203]
miR‐29a‐3p	Downregulated	PTEN	miR‐29a‐3p ↑→ PTEN ↓→ p‐AKT↑, p‐mTOR↑, Bax↓, Bcl‐2↑, Caspase‐3↓	Promote apoptosis	[204]
miR‐29b‐3p	Downregulated	PTEN	miR‐29b‐3p ↑→ PTEN ↓→ p‐AKT↑, p‐mTOR↑	Promote apoptosis	[205]
miR‐212‐3p	Downregulated	PTEN	miR‐212‐3p ↑→ PTEN ↓→ p‐AKT↑, p‐mTOR↑, Bcl‐2↑, Caspase‐3↓	Promote apoptosis	[206]
miR‐494	Downregulated	PTEN	miR‐494 ↑→ PTEN ↓→ p‐AKT↑, p‐mTOR↑, Bax↓, Bcl‐2↑, Caspase‐3↓	Promote apoptosis	[207]
miR‐21	Downregulated	PTEN	miR‐21 ↑→ PTEN ↓→ p‐AKT↑, p‐mTOR↑, Caspase‐3↓, IL‐6↓, TNF‐α↓, IL‐1β↓, IL‐10↑,	Promote apoptosis and inflammation	[208]
miR‐17	Upregulated	PTEN	miR‐17 ↑→ PTEN ↓→ PI3K↑, AKT↑, mTOR↑, NF↓, GFAP↑	Promote axon regeneration and suppress glial scar formation	[209]
miR‐421‐3p	—	mTOR	miR‐421‐3p ↑→ mTOR ↓→ Beclin‐1↑, LC3II↑, p62↓, Bax↓, Bcl‐2↑, Caspase‐3↓	Activate autophagy and anti‐apoptosis (miR‐421‐3p↑)	[210]
miR‐99b‐5p	Upregulated	mTOR	miR‐99b‐5p ↑→ mTOR↓	Anti‐apoptosis	[211]
*lncRNAs*					
GAS5	Upregulated	miR‐93 → PTEN	GAS5 ↓→ miR‐93 ↑→ PTEN ↓→ IL‐6↓, TNF‐α↓, IL‐1β↓, IL‐10↑, Bax↓, Bcl‐2↑, Caspase‐3↓	Promote apoptosis and inflammation	[213]
ZFAS1	Upregulated	miR‐1953 → PTEN	ZFAS1 ↓→ miR‐1953 ↑→ PTEN ↓→ p‐PI3K↑, p‐AKT↑, Bax↓, Bcl‐2↑, Caspase‐3↓, IL‐1β↓, IL‐6↓, TNF‐α↓	Promote apoptosis and inflammation	[214]
PTENP1	Upregulated	miR‐19b and miR‐21 → PTEN	PTENP1 ↓→ miR‐19b↑, miR‐21 ↑→ PTEN↓	Promote apoptosis	[215]
XIST	Upregulated	miR‐494→ PTEN	XIST ↓→ miR‐494 ↑→ PTEN ↓→ p‐AKT↑, p‐mTOR↑, Caspase‐3↓	Promote apoptosis	[216]
*circ RNAs*					
circ_0000962	Downregulated	miR‐302b‐3p	circ_0000962 ↑→ miR‐302b‐3p ↓→ PI3K↑, p‐AKT↑, NF‐κB↓, IL‐1β↓, IL‐6↓, TNF‐α↓, IL‐18↓	Anti‐apoptosis and anti‐inflammatory	[219]

miRNAs are highly conserved ncRNAs, approximately 22 nucleotides in length, which bind to the 3′‐untranslated regions (3′‐UTRs) of mRNAs through complementary base pairing. Such interactions inhibit protein synthesis through mRNA degradation (caused by complete base pairing) or transient translation arrest (caused by incomplete base pairing). A single miRNA can target multiple mRNAs and bind to multiple miRNAs.^197^ Many miRNAs have been shown to indirectly regulate the PI3K/AKT signalling pathway by affecting the expression patterns of downstream target genes; they directly regulate various molecules in the PI3K/AKT signalling pathway to affect SCI. In a rat model of SCI, miR‐21‐5p overexpression activates the PI3K/AKT pathway, inhibits the expression of proapoptotic proteins (Bax, caspase‐3 and caspase‐9) and inflammatory factors (IL‐8, IL‐1β, IL‐6 and TNF‐α), promotes the expression of the anti‐apoptotic protein Bcl‐2 and reduces neuronal cell apoptosis and inflammation.^198^ Sirtuin 1 is a NAD^+^‐dependent deacetylase with important roles in the regulation of apoptosis and inflammation. The expression of miR‐138‐5p increases after SCI; miR‐138‐5p can directly bind to the 3′‐UTR of sirtuin 1. miR‐138‐5p inhibition has been shown to reduce apoptosis and inflammation by upregulating sirtuin 1, thereby decreasing PTEN expression and activating AKT.^199^ Specificity protein 1 (SP 1) and Rho‐associated kinase (ROCK) are both involved in the regulation of nerve cell apoptosis and inhibition of axon regeneration. Wang et al. found that the expression of miR‐135a‐5p decreased after SCI, while miR‐135a‐5p overexpression targeted the 3′‐UTR of SP 1 at positions 339–345, the 3′‐UTR of ROCK1 at positions 706–713 and the 3′‐UTR of ROCK2 at positions 570–576 to inhibit the expression of these proteins; these changes activated the AKT/GSK3β signal axis, inhibited nerve apoptosis and promoted axon regeneration.^200^ Cannabinoid receptor 1 is a G protein‐coupled receptor that is widely found in nerve cells. Rap1 is a small Ras‐like GTPase that regulates cell proliferation, survival and apoptosis through various pathways. Zhang et al. showed that miR‐338‐5p inhibits cannabinoid receptor 1, thereby activating Rap1; this interaction ultimately prevents nerve cell apoptosis by activating the PI3K/AKT pathway.^201^ Cytoplasmic polyadenylation element binding protein 1 (CPEB1) is involved in cell oxidative stress, inflammation and apoptosis. Wei et al. found that CPEB1 expression increased after SCI, while miR‐122‐5p expression decreased. In addition, miR‐122‐5p overexpression targeted CPEB1 and inhibited SCI by activating the PI3K/AKT signalling pathway.^202^ CD47, also known as integrin‐associated protein, belongs to the immunoglobulin superfamily and participates in multiple cellular activities, including apoptosis, proliferation and migration. Disorders of miR‐34a pathways are related to neuron development; they can downregulate the expression levels of CD47 to activate the PI3K/AKT signalling pathway, thereby inhibiting SCI‐induced apoptosis in spinal cord neurons.^203^ PTEN is an inhibitor of the PI3K/AKT signalling pathway. Many miRNAs can affect the PI3K/AKT signalling pathway by regulating PTEN; these miRNAs include miR‐29a‐3p,^204^ miR‐29b‐3p,^205^ miR‐212‐3p,^206^ miR‐494^207^ and miR‐21.^208^ Overexpression of these miRNAs after SCI inhibits PTEN expression through the PTEN/AKT signalling axis, thus decreasing apoptosis and inflammation; it also promotes the recovery of nerve function in SCI animals. Furthermore, miR‐17 has been shown to promote the formation of glial scars and inhibit axon regeneration in the chronic phase of SCI by targeting PTEN to activate the PI3K/AKT pathway.^209^ mTOR is a downstream molecule of the PI3K/AKT signalling pathway; it prevents autophagy and promotes apoptosis. After SCI, miR‐421‐3p overexpression can inhibit mTOR expression by binding to the 3′‐UTR of mTOR, thereby promoting autophagy and inhibiting neuronal apoptosis.^210^ However, miR‐99b‐5p has been shown to inhibit neuronal apoptosis and inflammation after SCI by inhibiting the expression of the target gene mTOR.^211^

lncRNAs are ncRNAs with >200‐nucleotide transcripts; they can participate in the regulation of protein‐coding genes at multiple levels, including epigenetic regulation, transcription regulation and post‐transcriptional regulation. In addition, some lncRNAs bind to miRNAs, thus acting as miRNA ‘sponges’ by sharing a common binding site with miRNAs and preventing miRNAs from binding to their target mRNAs.^212^ After SCI, many pathways, including lncRNAs, participate in regulation of the PI3K/AKT signalling pathway. Many lncRNAs regulate PTEN expression through intermediate miRNAs, which then regulate the PI3K/AKT signalling pathway. The lncRNA growth arrest‐specific 5 is upregulated after SCI in mice; this lncRNA has been shown to negatively regulate miR‐93 and upregulate PTEN expression, which inactivates the PI3K/AKT pathway, thereby inducing apoptosis and inflammation after SCI.^213^ Chen et al.^214^ reported that the lncRNA zinc finger antisense 1 binds to miR‐1953, upregulates the target gene PTEN and inhibits the PI3K/AKT pathway, thus promoting SCI. Knockdown of ZFAS1 inhibits apoptosis and inflammation. The lncRNA phosphatase and tensin homologue pseudogene 1 (PTENP1) is the pseudogene of PTEN; it promotes PTEN expression by binding to miRNAs that target PTEN. Wang et al. showed that the inhibition of PTENP1 can simultaneously regulate miR‐19b and miR‐21, thereby inhibiting PTEN expression.^215^ The lncRNA X‐inactive specific transcript (XIST) upregulates the expression level of miR‐494. Overexpression of miR‐494 targets the 3′‐UTR of XIST to inhibit PTEN expression and activate the PI3K/AKT signalling pathway, thereby inhibiting SCI‐induced apoptosis in neurons.^216^

circRNAs are single‐stranded transcripts with a covalent closed loop structure. The lengths of circRNAs range from tens to thousands of base pairs. Compared with linear RNAs, circRNAs have a more stable structure. The circRNAs regulate gene transcription, participate in protein translation and regulate RNA‐binding proteins. In addition, similar to lncRNAs, circRNAs act as ‘sponges’ for miRNAs, thus inhibiting miRNA functions.^217^, ^218^ He et al.^219^ found that the expression of circ_0000962 decreased after SCI; the 3′‐UTR region of circ_0000962 exhibited potential homology with the miR‐302b‐3p sequence. In an in vitro model of SCI, overexpression of circ_0000962 inhibited miR‐302b‐3p, which induced the expression of both PI3K and p‐AKT proteins, while inhibiting the expression of NF‐κB protein. The circ_0000962 has also been shown to reduce the expression levels of inflammatory factors, such as TNF‐α, IL‐1β, IL‐6 and IL‐18. Therefore, overexpression of circ_0000962 after SCI has an anti‐inflammatory effect.

### 5.2 Biomolecules

In addition to the receptor tyrosine kinase, G protein‐coupled receptor and Ras families, various cellular molecules can regulate the PI3K/AKT signalling pathway after SCI (Table 2). Nerve growth factor (NGF) is an important member of the neurotrophic factor family with important roles in neuron development, axon growth, neurotransmitter synthesis and apoptosis.^220^ Xia et al.^221^ showed that NGF inhibited oxidative stress‐induced apoptosis in an in vitro model of SCI. NGF significantly activated the PI3K/AKT signalling pathway, promoted the expression of the anti‐apoptotic protein Bcl‐2 and inhibited the expression of the proapoptotic proteins Bax and caspase‐3. Furthermore, the use of gelatin nanostructured lipid carrier encapsulated NGF to treat SCI rats inhibited the endoplasmic reticulum (ER) injury‐induced apoptosis, increased neuron survival and improved functional recovery by activating the PI3K/AKT/GSK‐3β pathway.^24^ Mesencephalic astrocyte‐derived neurotrophic factor (MANF) is a conserved dopamine neurotrophic factor family that is widely expressed in various tissues, mainly in the ER and the Golgi complex.^222^ MANF has protective effects on nerve cells and can be used to treat SCI in rats. Mesencephalic astrocyte‐derived neurotrophic factor treatment in SCI rats upregulates the expression level of Bcl‐2 and reduces the levels of Bax and caspase‐3. In addition, it improves motor function, spinal cord water content and blood‐spinal cord barrier destruction. These effects may be mediated by the activation of AKT and murine double minute‐2, an oncogene with ubiquitination activity.^223^

**TABLE 2:** Biomolecules regulate the PI3K/AKT signalling pathway after SCI

Biomolecules	Mechanisms	Functions	References
NGF	NGF ↑→ p‐PI3K↑, p‐AKT↑, Bcl‐2↑, Bax↓, Caspase‐3↓	Anti‐apoptosis and anti‐inflammatory	[24, 221]
MANF	MANF ↑→ p‐AKT↑, MDM2↑, Bcl‐2↑, Bax↓, Caspase‐3↓	Anti‐apoptosis	[222]
FGF10	FGF10 ↑→ FGFR2↑, p‐AKT↑, TLR4↓, p‐I*κ*B *α*↓, NF‐*κ* B↓, Iba‐1↓, IL‐6↓, TNF‐ *α*↓, Bcl‐2↑, Bax↓, Caspase‐3↓	Anti‐apoptosis and anti‐ inflammatory	[225, 226]
bFGF	bFGF ↑→ p‐AKT↑, p‐mTOR↑, p‐GSK3β↑, LC3 II↓, p62↑, CHOP↓, GRP78↓, Caspase‐12↓	Anti‐apoptosis and anti‐autophagy	[227, 228]
IGF‐1	IGF‐1 ↑→ p‐AKT↑, P‐mTOR↑, LC3 II↓, Caspase‐9↓	Anti‐apoptosis and anti‐overautophagy	[113, 230]
GOT1/OxAc	GOT1↑, OxAc ↑→ Glu↓, P‐AKT↑, IL‐6↓, TNF‐*α*↓, *IL‐1β*↓	anti‐inflammatory	[231]
EST	EST ↑→ GPR30↑, p‐AKT↑, Bcl‐2↑, Bax↓, Caspase‐3↓, Caspase‐8↓	Anti‐apoptosis	[232, 233]
Nrg‐1β1	Nrg‐1β1 ↑→ p‐AKT↑, Bax↓, Bak↓, Caspase‐3↓, Caspase‐9↓	Anti‐apoptosis	[234]
Ch ABC	Ch ABC ↑→ p‐AKT↑, CSPG↓, Caspase‐3↓	Anti‐apoptosis, promote axon regeneration and suppress glial scar formation	[25]
MT	MT ↑→ p‐PI3K↑, p‐AKT↑, PDK1↑, PTEN↓	Anti‐apoptosis	[235]
BAMBI	BAMBI ↑→ TGF‐β↓, mTOR↓, NF‐κB↓, LC3II↑, Beclin‐1↑, IL‐6↓, IL‐1β↓	Promote autophagy and anti‐inflammatory	[236]
PHB 1	PHB1 ↑→ p‐PI3K↑, p‐AKT↑, NF‐κB↓, Bax↓, Bcl‐2↑, Caspase‐3↓, CHOP↓, GRP78↓	Anti‐apoptosis	[237]
ERp 29	ERp 29 ↑→ PI3K↑, Caspase‐3↓	Anti‐apoptosis	[238]

Fibroblast growth factor (FGF) is a family of cell signalling molecules released by various tissues; it has a wide range of biochemical and biological properties. FGF10, a member of the FGF family, has important roles in the regulation of several biological functions, such as morphogenesis, proliferation and the inhibition of apoptosis.^224^ After SCI, FGF10 expression is increased and the PI3K/AKT signalling pathway is activated by the FGF receptor 2 (FGFR2) to mediate diverse biological responses. Treatment of SCI rats with exogenous FGF10 reduced the expression levels of TLR4, p‐IκBα and NF‐κB (p65); it also inhibited nuclear translocation. In addition, the release of proinflammatory cytokines (e.g., Iba‐1, IL‐6 and TNF‐α) from the microglia and macrophages was reduced, whereas inhibition of the PI3K/AKT signalling pathway or knockdown of FGFR2 eliminated these treatment effects. In an in vitro SCI model of oxidative stress‐induced cell injury, FGF10 inhibited apoptosis by activating the PI3K/AKT signalling pathway.^225^, ^226^ Basic FGF (bFGF), also known as FGF2, is widely expressed in the nervous system and has multiple effects. Zhang et al.^227^ found that the injection of bFGF into injured spinal cord areas was sufficient to promote axon regeneration and functional recovery. In addition, bFGF treatment promoted the phosphorylation of AKT and mTOR; it inhibited LC3II expression. The levels of ubiquitinated proteins were also significantly reduced. Therefore, the therapeutic effect of bFGF constitutes inhibition of excessive autophagy by activating the PI3K/AKT/mTOR pathway and enhancing the clearance of ubiquitinated proteins. Additionally, bFGF promotes the expression of growth‐associated protein 43 (GAP43) through the activation of PI3K/AKT/GSK‐3β; it also inhibits the ER stress‐induced apoptosis response proteins (CHOP, GRP78 and caspase‐12), thus promoting recovery after SCI.^228^

IGF‐1 is an important neurotrophic factor that regulates neuron growth, dendritic branching and synapse formation.^229^ IGF‐1 expression levels decrease after SCI. Intravenous injection of IGF‐I in SCI rats causes PI3K/AKT/mTOR activation, upregulation of the expression levels of p‐AKT and p‐mTOR, and LC3II inhibition. In addition, IGF‐I injection weakens the activation of caspase‐9, increases the expression of the anti‐apoptotic protein Bcl2, inhibits apoptosis and excessive autophagy after SCI, and promotes the recovery of nerve function.^113^, ^230^

After SCI, cell necrosis leads to increased levels of extracellular glutamate, which promotes inflammation, glial scar formation and neuronal cell death. Blood glutamate scavengers, such as the blood resident enzyme glutamate‐oxaloacetate transaminase 1 and its co‐substrate oxaloacetate, remove excess glutamate from the spinal cord tissue to the systemic blood circulation; this process facilitates the neutralization of glutamate and its transamination into 2‐ketoglutarate. In blood glutamate scavenger‐treated SCI mice, the blood glutamate level was significantly reduced; moreover, p‐AKT protein expression was increased, the expression levels of proinflammatory factors (TNF‐α, IL‐1β, IL‐6 and iNOS) were significantly decreased, and the expression level of caspase‐3 in nerve cells at the lesion site was reduced.^231^

Oestrogen is a class of steroid compounds with a wide range of biological activities. Because of its strong antioxidant, anti‐inflammatory and anti‐apoptotic properties, oestrogen has been widely studied to investigate its potential therapeutic role for nerve injury after SCI. Oestrogen and its agonists can reduce PTEN expression and activate AKT to inhibit apoptosis after SCI.^232^, ^233^

In addition to the previously mentioned biomolecules, many other biomolecules can promote recovery after SCI by activating the PI3K/AKT signalling pathway. Neuromodulin‐1β1,^234^ ChABC,^25^ melatonin,^235^ activin membrane binding inhibitor,^236^ prohibitin 1^237^ and ER protein 29^238^ can inhibit inflammation, apoptosis and glial scar formation after SCI by activating the PI3K/AKT signalling pathway; they can promote nerve cell survival and axon regeneration to improve recovery after SCI.

### 5.3 Drugs

Several drugs have been found to exhibit neuroprotective effects in SCI (Table 3). Metformin is a traditional hypoglycemic drug that reduces the blood sugar level by inhibiting the production of liver glucose and increasing peripheral glucose consumption.^239^ It is commonly used in the treatment of type 2 diabetes and other metabolic syndromes. There is increasing evidence that metformin also has protective effects against various neurological diseases. Wang et al.^240^ showed that metformin use after SCI promoted the expression of ace‐tubulin and microtubule‐associated protein 2 in the spinal cord, thereby enhancing axon regeneration. Metformin treatment can increase the p‐AKT and Bcl‐2 levels, while inhibiting the SCI‐induced increases in caspase‐3 and Bax expression levels. Furthermore, metformin can treat SCI by regulating autophagy and apoptosis. Metformin can significantly inhibit mTOR expression after SCI and promote the expression of key autophagy proteins (Beclin‐1 and LC3II).^241^

**TABLE 3:** Drugs regulate the PI3K/AKT signalling pathway after SCI

Drugs	Mechanisms	Functions	References
Metformin	Metformin → p‐AKT↑, mTOR↓, Bax↓, Bcl‐2↑, Caspase‐3↓, Beclin1↑, LC3B↑, p62↓	Anti‐apoptosis, promote axon regeneration and autophagy	[240, 241]
FTY720	FTY720 → PTEN↓, p‐AKT↑, Bax↓, Bcl‐2↑	Anti‐apoptosis	[243]
DHA	DHA → p‐AKT↑	Anti‐apoptosis	[245]
Baicalin	Baicalin → p‐PI3K↑, p‐AKT↑, NF‐κB↓, Bax↓, Bcl‐2↑, Caspase‐3↓	Anti‐apoptosis	[23]
Euxanthone	Euxanthone → PI3K↑, p‐AKT↑, TLR4↓, NF‐κB↓, TNF‐α↓, IL‐6↓, IL‐12↓, IL‐1β↓	Anti‐apoptosis and anti‐inflammatory	[22]
Protocatechuic Aldehyde	Protocatechuic aldehyde → p‐PI3K↑, p‐AKT↑, PTEN↓, Bax↓, Caspase‐3↓, Caspase‐9↓	Anti‐apoptosis	[246]
Salvianolic acid B	Salvianolic acid B → p‐PI3K↑, p‐AKT↑, GSK3β↑, Bax↓, Caspase‐3↓, Caspase‐9↓, Bcl‐2↑	Anti‐apoptosis	[247]
Tetrahydrocurcumin	Tetrahydrocurcumin → p‐AKT↑, FOXO4↑, NF‐κB↓, TNF‐α↓, IL‐6↓, IL‐1β↓, Bax↓, Caspase‐3↓	Anti‐apoptosis and anti‐inflammatory	[248]
Icariin	Icariin → p‐AKT↑, CHOP↓, Caspase‐9↓, Bax↓, Bcl‐2↑, Caspase‐12↓, Caspase‐3↓	Anti‐apoptosis	[249]
Thymoquinone	Thymoquinone → PI3K↑, p‐AKT↑, TNF‐α↓, IL‐6↓, IL‐18↓, IL‐1β↓, Caspase‐3↓, Caspase‐9↓	Anti‐apoptosis and anti‐inflammatory	[181]
Isopsoralen	Isopsoralen → p‐PI3K↑, p‐AKT↑, ERα↑, Bax↓, Bcl‐2↑	Anti‐apoptosis	[250]
Palmitoylethanolamide	Palmitoylethanolamide → p‐AKT↑, mTOR↑, p‐70S6K↑, Beclin‐1↓, p62↑, LC3‐II↓	Anti‐overautophagy	[112]
Proanthocyanidins	Proanthocyanidins → p‐PI3K↑, p‐AKT↑, Bax↓, Caspase‐3↓, Caspase‐9↓, Bcl‐2↑	Anti‐apoptosis	[251]

FTY720 is a functional antagonist of sphingosine 1‐phosphate receptor‐1; it is widely used as an immunosuppressant in various diseases.^242^ In an in vitro model of SCI, exosomes derived from neural stem cells loaded with FTY720 were found to downregulate PTEN expression in spinal microvascular endothelial cells, while promoting p‐AKT expression. These effects reduced apoptosis by regulating the PTEN/AKT signalling pathway. FTY720 treatment can improve vascular endothelial function, reduce apoptosis and spinal cord edema and improve systemic function and behaviour after SCI by regulating the PTEN/AKT signalling pathway.^243^

The polyunsaturated fatty acid docosahexaenoic acid is a highly unsaturated fatty acid that is essential for brain nutrition. The use of docosahexaenoic acid prevents the deposition of cholesterol on the blood vessel wall and reduces atherosclerosis and coronary heart disease.^244^ It also has therapeutic potential in the treatment and prevention of neurological deficits after SCI. In a cell model of SCI, docosahexaenoic acid treatment stimulates AKT phosphorylation and inhibits apoptosis, whereas PI3K inhibitors prevent the beneficial effects of docosahexaenoic acid.^245^

In addition, various natural compounds extracted from plants activate the PI3K/AKT signalling pathway and participate in the treatment of SCI. In both in vitro and in vivo models of SCI, baicalin,^23^ euxanthone,^22^ protocatechuic aldehyde,^246^ salvianolic acid B,^247^ tetrahydrocurcumin,^248^ icariin,^249^ thymoquinone,^181^ isopsoralen,^250^ palmitoylethanolamide^112^ and proanthocyanidins^251^ have been shown to inhibit inflammation or neuronal apoptosis by regulating the PI3K/AKT signalling pathway. Table 3 summarizes the modulators of the PI3K/AKT signalling pathway and their potential mechanisms and functions.

## 6 CONCLUSION AND PERSPECTIVE

Secondary injury after SCI is a global problem. There is a need to understand the pathological processes involved in secondary SCI, then identify potential therapeutic targets to prevent the progression of secondary injury. This review summarized the pathological processes and mechanisms underlying inflammation, cell death and glial scar formation in secondary injury. Early apoptosis and autophagy after SCI protect the body against injury; a prolonged inflammatory response leads to the accumulation of proinflammatory factors and excessive apoptosis, as well as autophagy in the surrounding normal nerve cells, thus aggravating SCI in the subacute stage of secondary injury. Initial glial scar formation in the subacute phase is a protective mechanism for SCI, which limits the spread of damage and inflammation. However, mature scar tissue in the chronic phase hinders axon regeneration and prevents the recovery of nerve function. Therefore, in the acute phase of SCI, surgical intervention and suppression of inflammatory responses may be needed. In the subacute phase, apoptosis, disruption of autophagic flow and excessive autophagy should be suppressed. In the chronic phase, glial scars can be ablated to promote axon regeneration. These strategies may be feasible for the treatment of SCI, but additional studies are necessary to compare the onset timings of acute, subacute and chronic phases.

The PI3K/AKT signalling pathway has an important role in the recovery of spinal cord function after secondary injury. Activation of the PI3K/AKT signalling pathway can inhibit the activation of immune cells and block the release of proinflammatory cytokines. In addition, this pathway can promote the polarization of macrophages to the anti‐inflammatory type and lead to increased secretion of anti‐inflammatory factors. The PI3K/AKT signalling pathway can also regulate the programmed death pathway. Furthermore, activation of this pathway inhibits apoptosis after SCI through effects on the extrinsic or intrinsic apoptosis pathway; it also inhibits excessive autophagy and cell death. However, inhibition of this pathway can promote autophagy, which leads to inhibition of apoptosis. This contradictory biological function may be explained by differences in time points, activators or inhibitors and cell types used in vitro experiments. Many studies have explored the biological effects of the activation or inhibition of the pathway at different time periods by knocking out or knocking in key molecules of the pathway. In addition, PI3K/AKT signalling pathway activation can promote the proliferation of spinal cord astrocytes, whereas inhibition of this pathway can reduce the formation of glial scars and promote recovery after SCI. Therefore, PI3K/AKT signalling pathway activation is involved in the subacute phase of secondary injury; inhibition of this pathway in the chronic phase may offer a strategy for the treatment of SCI.

In summary, this review discussed the pathological processes of inflammation, cell death and glial scar formation in secondary SCI, as well as the potential therapeutic effects of the PI3K/AKT signalling pathway. Additional studies are needed to improve the general understanding of the pathogenesis of secondary SCI and the complex mechanism of action of the PI3K/AKT pathway, thus establishing a comprehensive theoretical basis for the treatment of SCI based on the PI3K/AKT pathway.

## AUTHOR CONTRIBUTIONS

Xuegang He, Yong Yang and Xuewen Kang conceived and designed the review. Xuegang He drafted the manuscript; Ying Li and Bo Deng assisted in the preparation of the charts; Aixin Lin, Guangzhi Zhang and Miao Ma edited and revised the manuscript; all authors read and approved the final version of the manuscript.

## CONFLICT OF INTEREST

The authors declare no competing interests.

## Data Availability

Data sharing not applicable to this article as no data sets were generated or analysed during the current study.
